# Long COVID Following Mild SARS-CoV-2 Infection: A Retrospective Study in a Portuguese Primary Health Care Unit

**DOI:** 10.7759/cureus.73655

**Published:** 2024-11-14

**Authors:** Andreia Barata, Manuela Sebastião, Nelma Sampaio, Luana Lima, Isa Cruz, Isabel Antunes

**Affiliations:** 1 Family Medicine, Primary Health Care Unit - USF Amatus, Castelo Branco, PRT

**Keywords:** complications, covid-19, long covid, post-covid-19 condition, sequelae

## Abstract

Introduction: Long COVID is a recent pathological entity. Its manifestations and impact on health and quality of life and, on a larger scale, at an economic and social level may be very significant, reflecting a challenge for the future. The family doctor plays a major role in the care of these patients and must be aware of this new reality. With this study, we aim to determine the prevalence of long COVID in the adult population registered in a Portuguese Primary Healthcare Unit, called USF Amatus, and identify the most frequent symptoms, its duration, and impact on the quality of life of individuals.

Methods: This is a retrospective study based on the analysis of the national database of COVID-19 (Trace COVID-19), selecting patients ≥ 18 years old registered in USF Amatus, diagnosed with mild COVID-19, in the period between March 2020 and March 2022. The selected patients were submitted to a telephone interview to fill a questionnaire adapted from the World Health Organization “Report Form for Post COVID Condition” to the Portuguese population by the Directorate-General of Health (DGS). The collected data were analyzed using statistical software (R Development Core Team; Vienna, Austria) and the package “ggplot2”.

Results: A total sample of 334 (56% female) was recruited for this study. The prevalence of long COVID was found to be 145 (43.4%) cases in the near months (≥ 3 months) following COVID-19 and 57 (17.1%) cases after two years. The most frequently reported symptoms were fatigue, persistent muscle pain, post-exercise malaise, memory loss, leg/arm weakness, and dizziness/lightheadedness. About 58 (17.4%) patients reported that their self-care capacity became worse after COVID-19. In the further evaluation of functional status, 56 (16.8%) patients reported that walking a distance of at least 1 km is more difficult nowadays, and 29 (8.7%) reported that performing household tasks became harder.

Conclusion: This study demonstrates the relevance of long COVID. The findings reveal a significant prevalence of symptomatic patients even two years after the infection with SARS-CoV-2. The most commonly reported symptom was fatigue. Other prevalent symptoms were neurocognitive, muscular, and physical exercise intolerance. It is also important to mention the relevant impact of long COVID on the quality of life of these patients. Further research is needed in order to better understand long COVID and, consequently, develop more directed and effective interventions for these patients, contributing to their well-being at an individual, familial, and social level.

## Introduction

COVID-19 is a multisystemic infectious, inflammatory, and thrombogenic disease of variable duration and clinical presentation [1,2]. Possible clinical presentations range from the absence of symptoms, nonspecific symptoms such as fever and myalgia, to severe respiratory and cardiovascular complications, including acute respiratory distress syndrome and multiorgan failure [2]. Scientific evidence has shown that these patients may develop complications of the disease, regardless of its severity, named long COVID [3]. It is estimated that the prevalence of post-COVID symptoms may range from 50% in the months near the acute infection up to 20% two years later [4].

According to the World Health Organization (WHO), long COVID is defined as a “post-COVID-19 condition that occurs in people with a history of probable or confirmed SARS-CoV-2 infection, usually three months from the onset of infection, with symptoms that last for at least two months and cannot be explained by an alternative diagnosis. Symptoms might be new onset following initial recovery from an acute COVID-19 episode or persist from the initial illness. Symptoms might also fluctuate or relapse over time” [5]. According to the National Institute for Health and Care Excellence (NICE), the term long COVID describes the signs and symptoms that continue or develop after acute COVID-19 and includes both symptomatic COVID-19 (from four to 12 weeks) and post-COVID-19 syndrome (12 weeks or more) [6]. The long COVID diagnosis is clinical [7].

The pathophysiology of this condition is not yet fully understood [4,8]. It may be due to an inflammatory state, dysregulation of antibody activation, continued viral activity, and organ damage as a reflection of the acute injury of the infectious phase. Other factors such as host genetic determinants and external factors such as vaccination should also be considered [3,8]. Thus, this syndrome has a multifactorial presentation. The symptoms may be related to the underlying medical condition, the presentation of the disease in the acute phase, and psychological effects associated with social isolation during the pandemic. The symptoms of long COVID are highly variable and fluctuating, and the most frequently described in the literature are fatigue, shortness of breath, neurocognitive (difficulty concentrating and cognitive dysfunction), and psychiatric symptoms (anxiety or depression) [7-10]. Their consequences and impact on the individual's health and quality of life and, to a greater extent, on an economic and social level, are not yet fully known, but they may be very significant and represent a challenge for the future [11,12]. Therefore, it is essential to make a correct and timely diagnosis and define proper guidelines for the evaluation and follow-up of these patients in order to anticipate and minimize the possible consequences of long COVID. The family doctor plays a major role in the care of these patients and must be aware of this new reality.

## Materials and methods

Study setting and sample size

This study was conducted in a Primary Health Care Unit, called USF Amatus, located in Castelo Branco, a city in the countryside of Portugal, approximately 200 km from the capital, Lisbon. The referral hospital is called Amato Lusitano and is located less than 1 km away from USF Amatus. Both units are part of the Local Health Care Unit (ULS) of Castelo Branco. USF Amatus is composed of a team of seven medical doctors, seven nurses, and five technical assistants and serves a population of 11,542 patients.

A retrospective study was carried out based on the analysis of the national database of COVID-19 patients (called Trace COVID-19) selecting the adults (age ≥ 18) registered in the USF Amatus, diagnosed with mild COVID-19, in the period between March 2020 and March 2022. A total population size of 2,514 individuals was obtained, and using the guidelines provided by Daniel and Cross [13], we determined a random sample (n = 334) that is representative of the total population. The sample was further stratified by gender, containing 187 women and 147 men. The selected patients were submitted to a telephone interview in order to fill out a questionnaire adapted from the WHO “Post-COVID-19 Case Report” to the Portuguese population by the Directorate-General of Health (DGS) [14]. The collected data were analyzed using statistical software (R Development Core Team, Vienna, Austria) and the package “ggplot2” [15,16].

The conclusions are derived from the percentages obtained, with the sample size ensuring that these population estimates lie within the 95% confidence interval, subject to a maximum margin of error of 4.99%.

Inclusion and exclusion criteria

Individuals who met the following criteria were included in this study: Patients had to be registered in the Primary Health Care Unit - USF Amatus and be 18 years of age or older. They must have been diagnosed with COVID-19 between March 2020 and March 2022. Eligible patients presented with asymptomatic or mild acute SARS-CoV-2 infection, with no need for hospitalization. Additionally, patients had to either develop new symptoms within three months or remain symptomatic after the acute infection, with the symptoms lasting for a minimum of two consecutive months. Moreover, these symptoms could not be explained by an alternative diagnosis.

Individuals who did not meet the inclusion criteria were excluded from the study. This included those under the age of 18, individuals who required hospitalization during the acute phase of COVID-19, patients with another diagnosis that could explain their presenting symptoms, and individuals who died before the study was conducted. Additionally, due to our method for obtaining the answers to the questionnaire, we excluded patients who did not respond to the telephone call after three attempts or who were unable to participate due to cognitive, speech, or other communication impairments, unless a reliable proxy was available to assist. In order to keep the same sample size (n = 334), we replace them by using the same guidelines [13].

**Figure 1:**
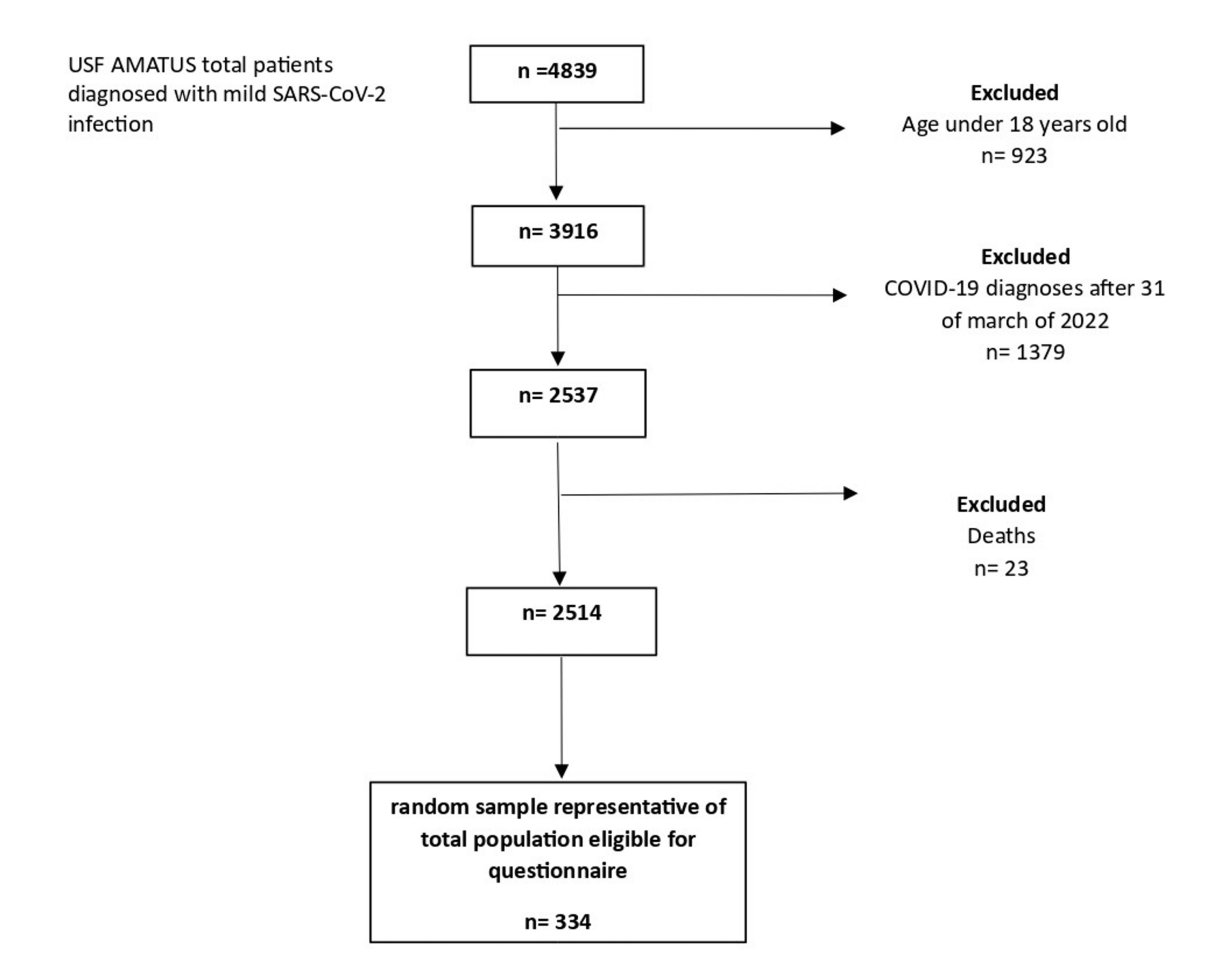
Study population flow chart. Number of participants eligible for questionnaire application

The study population flow chart is shown in Figure 1.

The total sample (n = 334) was composed of 187 males and 147 females. Those patients were submitted to a telephone interview, during June and July of 2024, and invited to answer a questionnaire adapted from the WHO “Case Report Form for Post COVID Condition” to the Portuguese population by the DGS for the follow-up of post-COVID-19 patients [14].

All the participants gave oral consent before entering the study.

The study protocol was approved by the Ethics Committee of the Local Health Care Unit (ULS) of Castelo Branco, on December 22, 2022.

## Results

**Figure 2:**
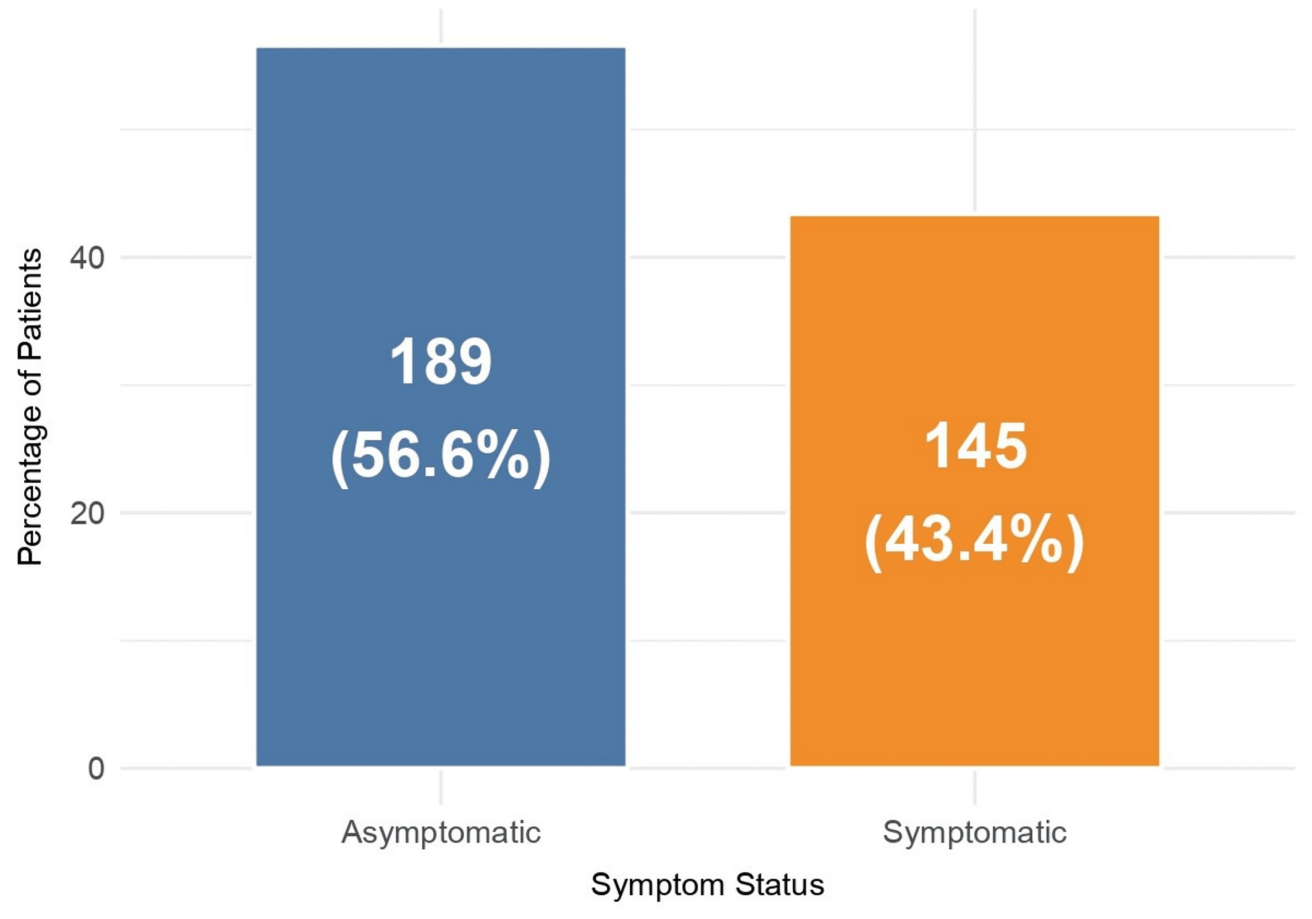
Percentage of asymptomatic and symptomatic patients three months after SARS-CoV-2 infection (n = 334)

As shown in Figure 2, from the total sample (n = 334), 145 (43.4%) patients remained symptomatic or developed symptoms three months after the mild SARS-CoV-2 acute infection. Those symptoms lasted at least two consecutive months and were not present prior to the COVID-19 infection. Additionally, 57 (17.1%) patients continued to report persistent symptoms two years after the acute disease.

**Figure 3:**
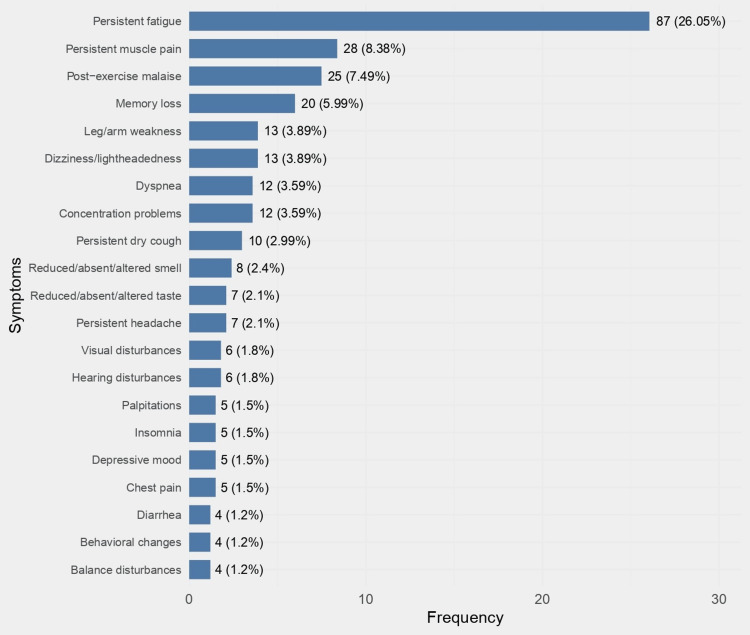
Frequency of persistent symptoms three months after SARS-CoV-2 infection that remained for at least two months (n = 145)

As seen in Figure 3, among the symptoms reported by the patients, fatigue was the most frequent, present in 87 (26.1%) individuals. Musculoskeletal and respiratory symptoms such as persistent muscle pain (8.4%), post-exercise malaise (7.5%), leg/arm weakness (3.9%), dyspnea (3.6%), and persistent dry cough (3%) were also mentioned. Neurocognitive symptoms, including memory loss (6%), dizziness/lightheadedness (3.9%), concentration problems (3.6%), and persistent headache (2.1%), were also frequent. Reduced/absent/altered smell and taste were also present in about 2% of the patients. Symptoms reported by less than 1% of patients are not shown in the graph. Other symptoms, not mentioned in the questionnaire, which a few patients also reported, were throat discomfort, post-nasal drip, sputum, hoarseness, and joint pain.

Among the 57 (17.1%) individuals who reported symptoms two years after SARS-CoV-2 infection, the most frequent were persistent fatigue (18%), post-exercise-malaise (5.7%), persistent muscle pain (5.7%), memory loss (5.7%), and dizziness/lightheadedness (3.4%). 

**Figure 4:**
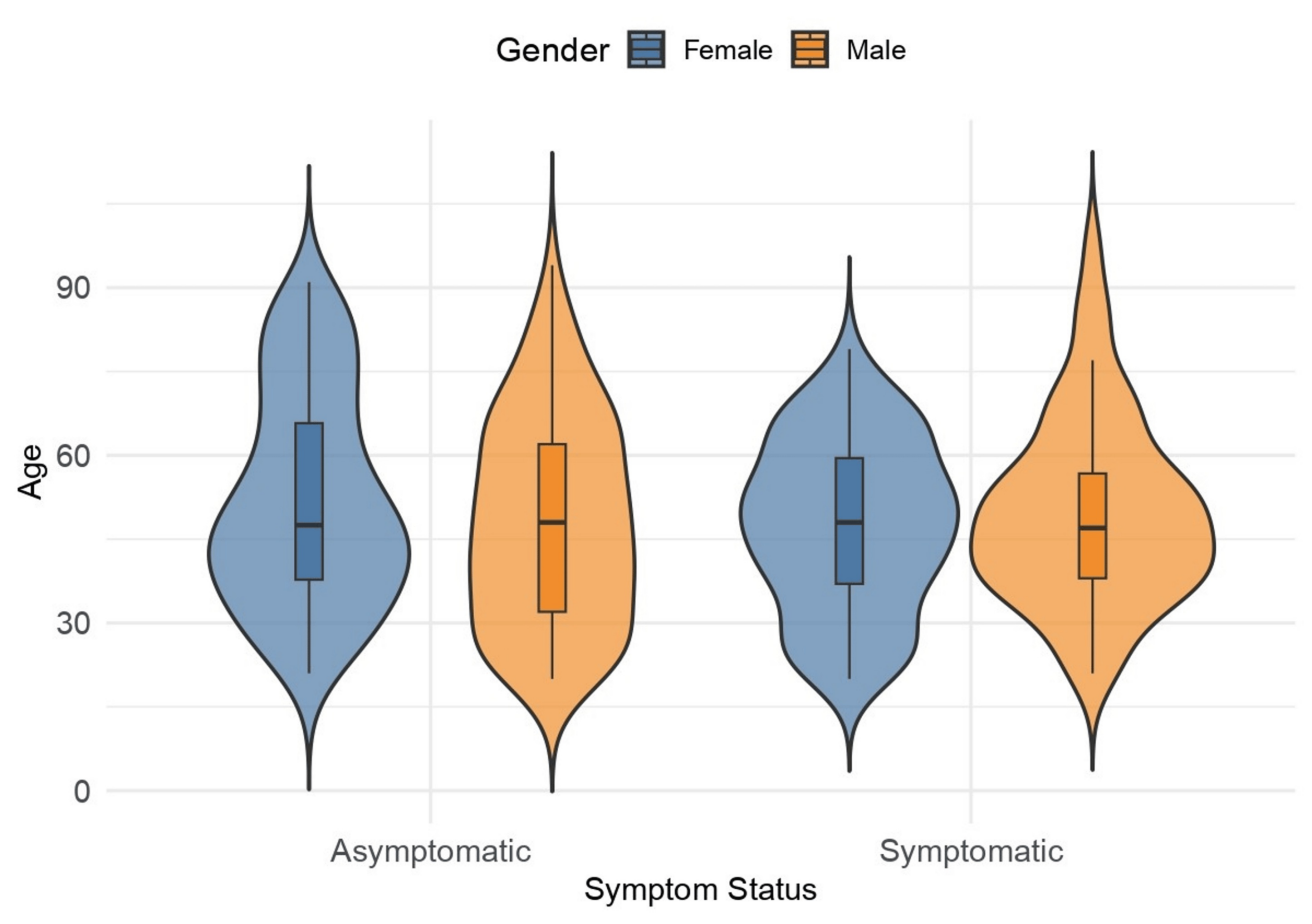
Violin plot of age distribution across gender and symptom status (n = 334)

The violin plot (Figure 4) shows that the age distribution of the patients appears to be centered around 30 and 60 years old. There is not a significant difference between the asymptomatic and symptomatic groups. The median is slightly above 40 years old in both groups. However, the distribution of asymptomatic patients (especially among women) appears to be more frequent in the 30-50 years old. In the asymptomatic patient group, men appear to have a more uniform age distribution. In symptomatic patients, men seem to have a more concentrated age distribution between 30 and 40 years old, while women have a more dispersed distribution and, consequently, greater age variability.

**Figure 5:**
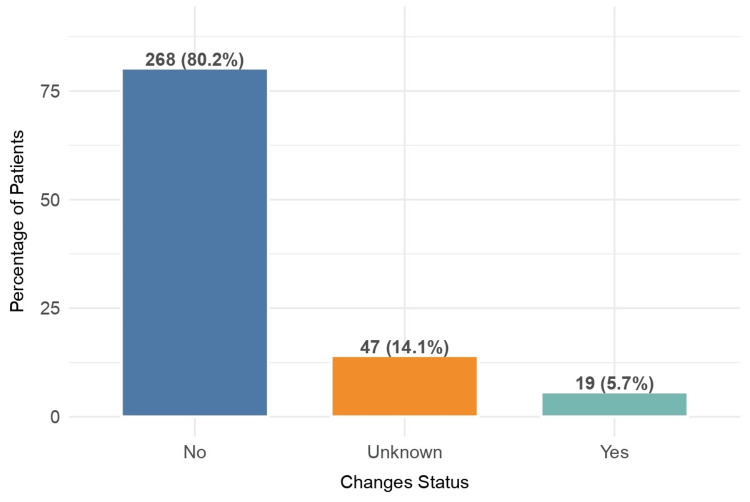
Changes in work/school duration compared to pre-COVID-19 levels (n = 334)

When asked about the impact on the work or school status after the SARS-CoV-2 infection, 268 of the patients (80.2%) reported that there was no impact on their labor life, and 19 (5.7%) patients said there was an impact after the COVID-19, which remains nowadays (Figure 5).

**Figure 6:**
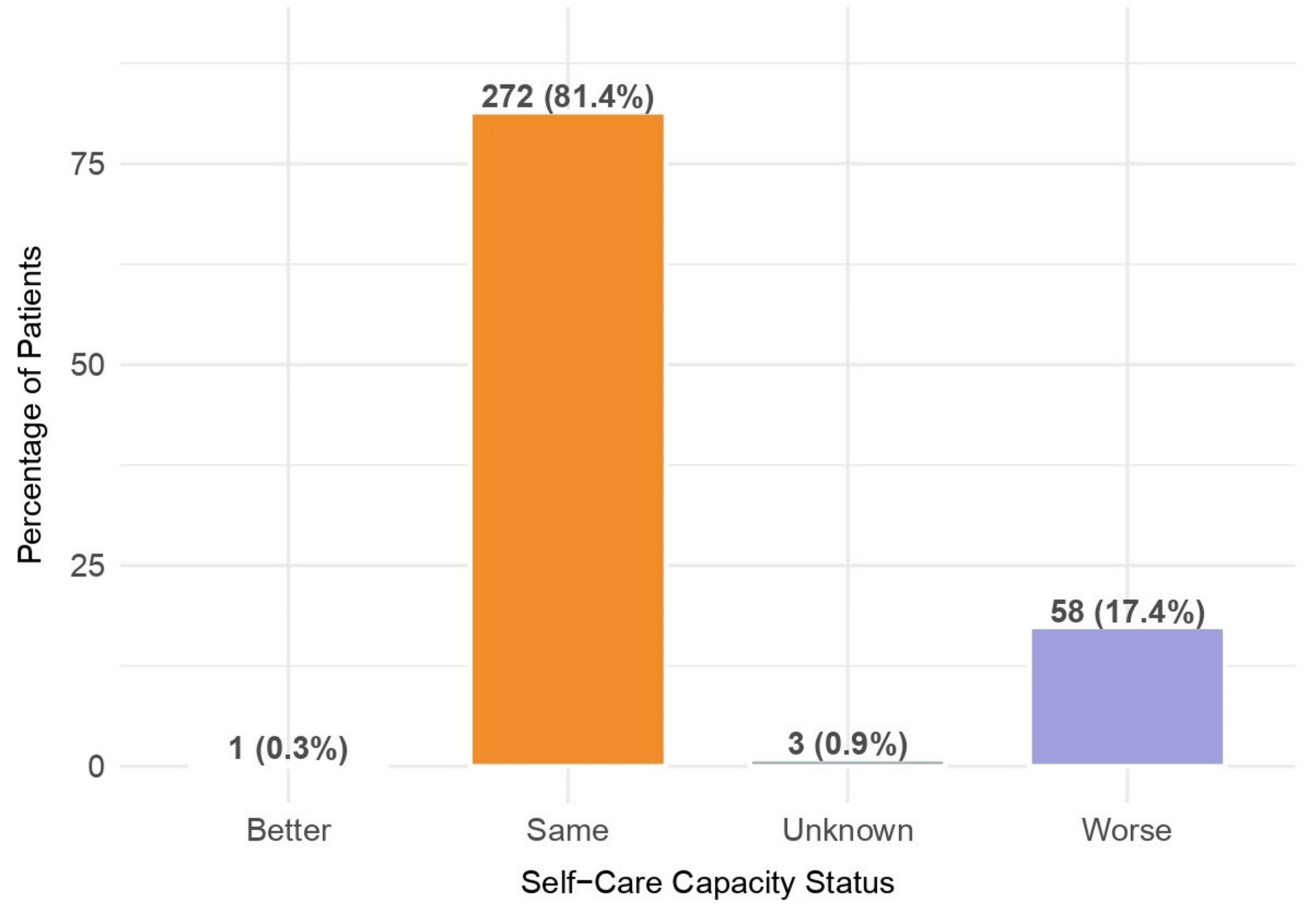
Self-care capacity compared to pre-COVID-19 levels (n = 334)

When asked if there were any changes in their self-care capacity after SARS-CoV-2 infection, 58 (17.4%) patients reported that it is worse nowadays than before COVID-19, and 272 (81.4%) reported that it remains the same (Figure 6).

**Figure 7:**
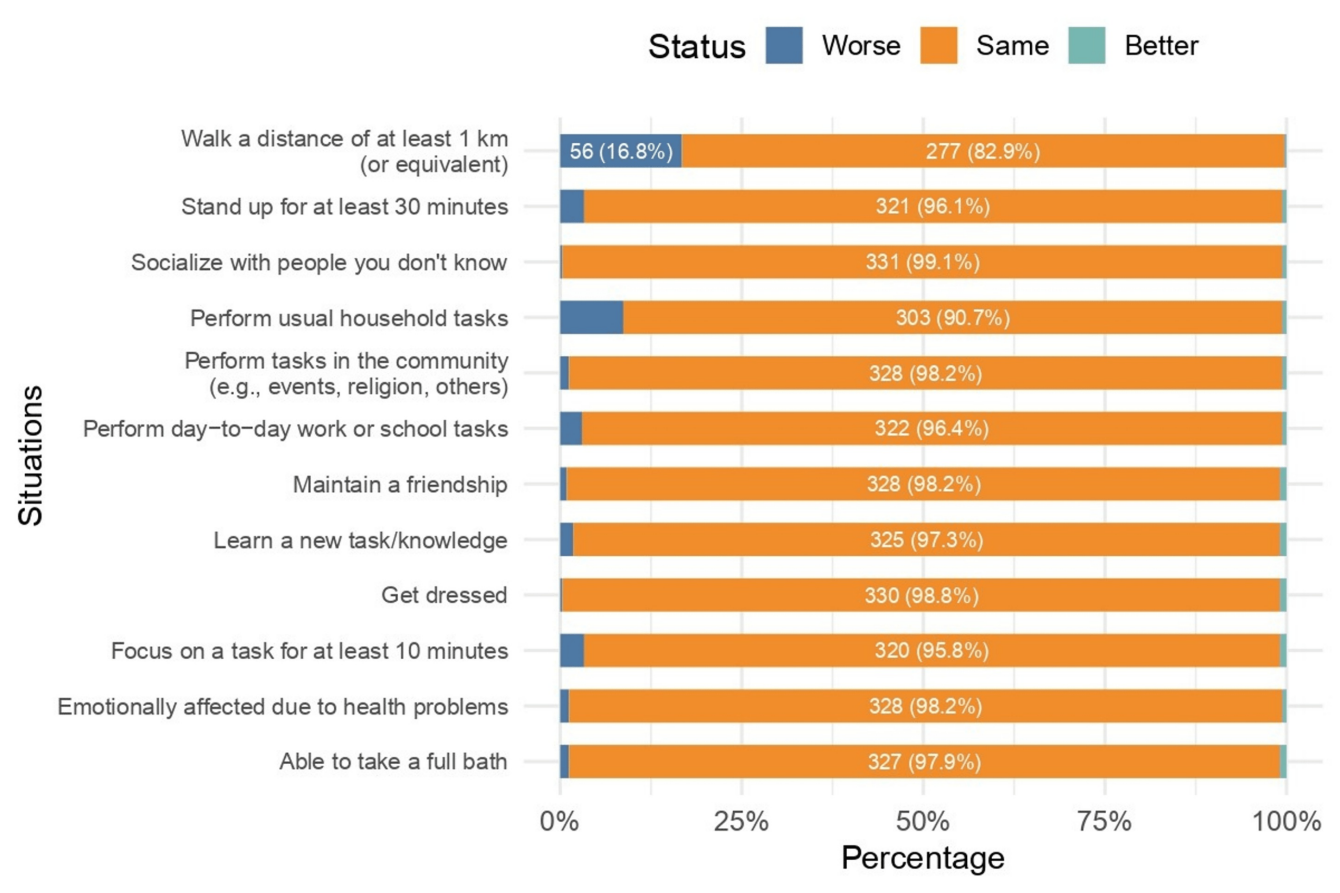
Level of difficulty in various situations over the past seven days (n = 334)

In the further evaluation of functional status in the present days (Figure 7), 56 (16.8%) individuals reported that walking a distance of at least 1 km (or equivalent) is worse now than before SARS-CoV-2 infection. About 3.9% mentioned that standing up for at least 30 minutes is harder nowadays. Additionally, 8.7% and 3.6% reported, respectively, that performing usual household tasks and performing day-to-day work or school tasks became more difficult. About 4.2% reported that the ability to focus on a task for at least 10 minutes is harder nowadays than previously due to COVID-19 disease.

## Discussion

A total of 334 participants (56% female) were recruited for this study. The mean age of the participants was 49.3 years.

The prevalence of long COVID was 43.4% in the near months (≥ 3 months) after COVID-19 disease, and 57 (17.1%) patients continued reporting persistent symptoms two years after the infection. These facts are in accordance with a study published by Fernández-de-las-Penas, which points to a significant portion of the population having persisting symptoms in the long term [4]. However, more research is needed to achieve more consistent results in the long-term evaluation of long COVID. It is important to emphasize that, in our study, patients had mild acute COVID-19 disease, without the need for hospitalization.

The most frequently reported symptoms were fatigue, persistent muscle pain, post-exercise malaise, memory loss, leg/arm weakness, and dizziness/lightheadedness. Fatigue was the most commonly reported symptom both as a short and long-term complication after the COVID-19 disease. The finding is in line with other scientific studies conducted in different countries [8-11,17,18].

There is not a significant difference between the asymptomatic and symptomatic groups in terms of age (centered around 30 and 60 years old). In men, long COVID is more prevalent between 30 and 40 years old, while women have a greater age variability. This may indicate that among women there is a greater range of ages among patients who present symptoms after COVID-19 disease [19].

It is important to mention that other symptoms, not considered in the questionnaire, were also reported by a few patients, mainly otolaryngological-related symptoms such as throat discomfort, post-nasal drip, sputum, and hoarseness. Patients reported that these symptoms were intermittent. It may be interesting to include these symptoms in the WHO standardized symptom checklist in the future. According to Mogitate, increasing reports indicate that long COVID symptoms, similar to chronic epipharyngitis may occur in patients after mild SARS-CoV-2 acute infection [20].

Long COVID is considered a very complex entity, with several clinical manifestations. According to Sapna et al., “the pathophysiological effects of SARS-CoV-2 virus extend beyond the initial phase of infection” [21]. In fact, several organ systems may be affected and, as this study reveals, that may occur even when the initial infection was mild. This may be due to a very complex interaction between several factors at an individual level. Sapna et al. pointed out viral persistence after the infection, immune dysregulation, and autoimmunity as key players in long COVID pathophysiology [21]. Thus, this helps us understand the better pathway to manage these patients.

As mentioned before, fatigue is the most prevalent symptom reported and can be very incapacitating [20]. In fact, about 17.4% of our patients reported that their self-care capacity after SARS-CoV-2 infection was compromised. According to Goodman et al., “disruptions to daily function explain some of the associations between chronic physical condition and worsened mental health outcome” [22]. Young and previously healthy individuals are also at risk [23].

In the further evaluation of functional status in the present days, 16.8% of the individuals reported that walking a distance of at least 1 km (or equivalent) is worse now than before the SARS-CoV-2 infection. About 3.9% mentioned that standing up for at least 30 minutes is harder nowadays.

Neurocognitive impairments are prevalent in this study and can have a significant impact on the quality of life. In fact, 4.2% reported that focusing on a task for at least 10 minutes is harder nowadays than previously due to COVID-19 disease. About 8.7% and 3.6% reported, respectively, that performing usual household tasks and performing day-to-day work or school tasks became more difficult.

Strengths and limitations

This study aims to fill the gap that exists in scientific research on long COVID patients, particularly in the population with mild SARS-CoV-2 acute infection. The literature suggests that up to 20% of the patients continue to experience symptoms two years after the acute COVID-19 infection. We aimed to evaluate those patients in order to assess the long-term sequelae and gain a better understanding of the long COVID entity. We used a standardized methodology during the process to achieve more statistically significant results. Patients with confirmed COVID-19 using the RT-PCR test were included, in the period between March 2020 and March 2022, accessing the data in the national database called “Trace COVID-19” and crossing that information with the patient medical history to select the eligible population for the study. We selected a random sample (n = 334), representative of the total population (n = 2,514) with the purpose of achieving a more consistent result and less bias. We had a high rate of acceptance for the questionnaire interview (only 24 did not want to participate), and with the aim of keeping the sample statistically representative, we replaced them by using the same guidelines in order to maintain the total sample (n = 334). Our study also has limitations. All data of the questionnaire were self-reported and thus may be prone to recall bias. Additionally, the individual patient comorbidities before COVID-19 disease might have an impact on the prevalence of long COVID. Thus, it is important to be cautious when generalizing these results to the general population.

## Conclusions

Long COVID is a medical condition that is still underestimated and undertreated. Most of the available studies refer to the evaluation of hospitalized patients during the acute phase of the disease or after hospitalization. However, it is extremely important to conduct studies on patients with mild presentation of the acute disease, as they are considered a vulnerable population for developing complications of the disease. This study highlights the relevance of long COVID in these patients, demonstrating a significant prevalence of symptoms even two years after mild SARS-CoV-2 infection.

The most commonly reported symptom was fatigue. It is important to mention that fatigue can be physical, cognitive, or emotional. Thus, the underlying mechanism of this symptom can be different among individuals with long COVID. Other relevant symptoms reported in this study were neurocognitive, musculoskeletal, and respiratory. It is also important to mention the significant impact of these symptoms on the patient's quality of life. A holistic and multidisciplinary approach is needed to manage these patients, with a focus on the symptomatic treatment, the etiological factors involved, and rehabilitation. Further research is needed in order to better understand long COVID and, consequently, develop more effective interventions for these patients that could lead to greater well-being at an individual, familial, and social level. By carrying out this study, we hope to contribute to a deeper and more detailed knowledge about long COVID.

## Figures and Tables

**Figure 8:**
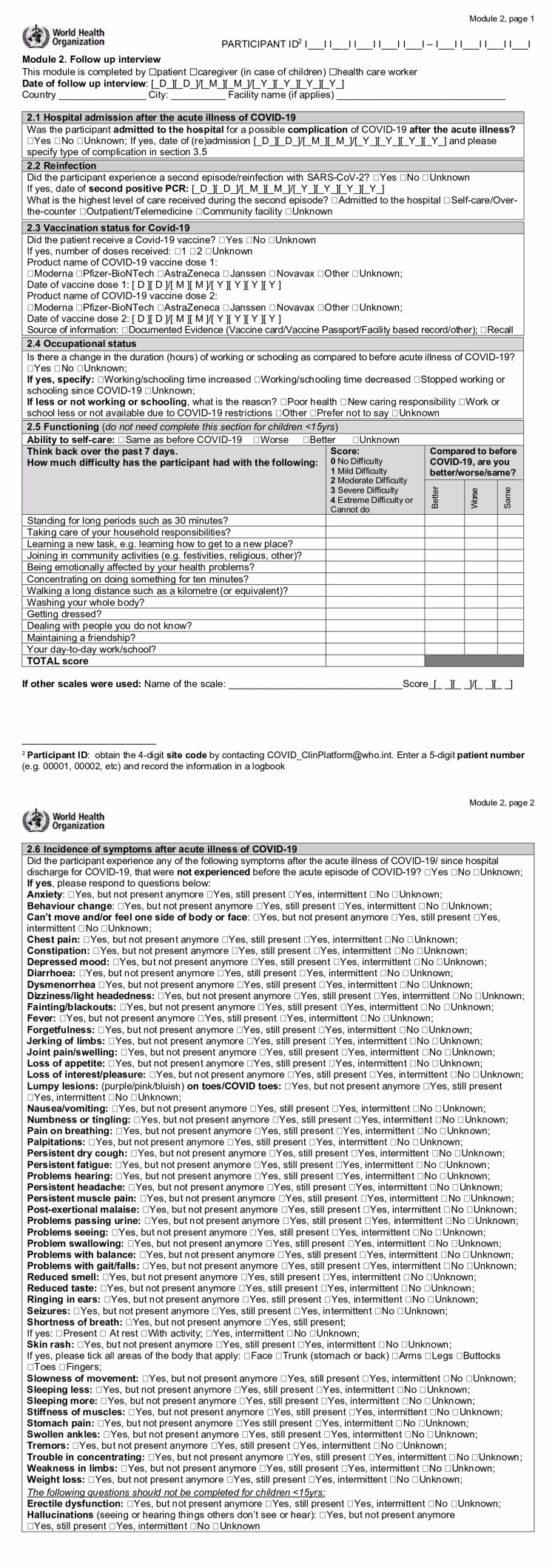
Case Report Form for Post COVID Condition License: CC BY-NC-SA 3.0 IGO [14]
